# Special Issue “Machine Learning and Bioinformatics in Human Health and Disease”—Chances and Challenges

**DOI:** 10.3390/ijms252312811

**Published:** 2024-11-28

**Authors:** Andreas Leiherer

**Affiliations:** Vorarlberg Institute for Vascular Investigation & Treatment (VIVIT), 6800 Feldkirch, Austria; andreas.leiherer@vivit.at

## 1. Introduction

Machine learning (ML) and bioinformatics are catalyzing a new era in biomedical research, enabling unprecedented insights into the complex systems that govern human health and disease. This Special Issue, “*Machine Learning and Bioinformatics in Human Health and Disease*”, showcases a collection of 11 articles—10 original research studies and 1 review—that underscore the profound impact of computational methods on advancing precision medicine. Integrating advanced ML techniques with biological sciences, these works highlight applications ranging from disease prediction and biomarker discovery to therapeutic innovation and treatment optimization. Collectively, this research reflects the transformative potential of ML and bioinformatics in making healthcare more predictive, preventive, and personalized.

## 2. Omics and Multi-Omics Integration for Precision Medicine

A prominent theme in this Special Issue is the use of omics and multi-omics—such as genomics, transcriptomics, and metabolomics—to understand disease mechanisms at a deeper level. Traditional single-data analyses, while valuable, often overlook the complex interactions that characterize many diseases. Integrating omics and multi-omics allows researchers to create more accurate, multidimensional models that can uncover novel biomarkers, improve diagnostic accuracy, and identify therapeutic targets more precisely.

Several studies in this Special Issue illustrate the promise of omics data for advancing precision medicine. By including big datasets and layering different data types, researchers are now better equipped to identify intricate biological patterns and correlations. This approach is especially powerful for multifactorial diseases, where genetic predisposition, environmental factors, and metabolic changes interact in complex ways. For example, Luo et al. leveraged a novel multi-omics integration approach referred to as TEMINET, in which mRNA, miRNA, and methylation data are combined to strengthen diagnostic predictions, illustrating the potential of multi-omics approaches for more accurate and early disease detection [1]. Similarly, Zhang et al.’s research is also noteworthy for their application of ML to transcriptome and single-cell RNA-seq datasets in regard to juvenile idiopathic arthritis, wherein the identification of four key genes with prognostic value for juvenile idiopathic arthritis, together with the pathways involved, provided critical insights into immune dysregulation in autoimmune disorders [2]. This study not only offers potential prognostic markers and therapeutic targets but also illustrates how ML can integrate complex transcriptomic data for a better understanding of immune-related pathologies. Meanwhile, Akshay et al. applied ML to transcriptomic data from patients with non-ulcerative bladder pain syndrome [3]. Their findings reveal distinct gene expression signatures that could serve as potential biomarkers, addressing a condition where reliable biomarkers have historically been scarce [3]. Similarly, regarding metabolic research, our group has applied ML techniques to metabolomic data to predict the risk of type 2 diabetes mellitus [4].

Collectively, these studies underscore the advantages of integrating diverse biological data layers to generate holistic, accurate insights into disease.

## 3. Unraveling Disease Mechanisms

Several studies in this Special Issue delve deeply into the genetic and molecular underpinnings of disease, shedding light on potential targets for novel treatments. By analyzing the interactions between genes, proteins, and other biomolecules, the authors of these studies use ML to discover hidden patterns that contribute to disease mechanisms.

For example, Farias et al. present a ML-learning-driven analysis of a competing endogenous RNA (ceRNA) network in metastatic clear-cell renal carcinoma [5]. Their research identifies an 11-gene signature associated with metastasis, revealing key molecular players that could serve as biomarkers or therapeutic targets. This approach illustrates how ML can reveal non-coding RNAs and regulatory molecules pivotal to disease progression [5]. In parallel, neurological disorders—characterized by their inherent complexity and diagnostic challenges—are addressed in the review by Aljarallah, Dutta, and Sait. Their work provides an in-depth analysis of how ML models are being applied to address these challenges by analyzing genetic and molecular pathway data [6]. Highlighting disorders such as Alzheimer’s disease (AD), Parkinson’s disease, autism spectrum disorder, and schizophrenia, this review examines the current progress in applying ML to detect early biomarkers and predict disease trajectories. However, as the authors note, translating ML discoveries into clinical settings remains a demanding task. Guaranteeing data quality, model interpretability, and validation across diverse patient populations are critical for ensuring that ML models are safe and reliable for clinical use [6]. Though this will be a future task in the ML field, this review’s insights on neurological disorders have provided valuable context, as it explores how ML-driven analyses can capture the complexity of these diseases by identifying subtle, non-linear relationships in large datasets. This idea resonates in the study by Zhao et al., who examined the connection between obesity and AD [7]. They integrated genetic and metabolic data to shed light on the etiology of AD, offering new insights into potential pathways and enabling earlier intervention. In addition, Blanot et al. aimed to explore the mode of action of intravitreal aflibercept injection in the pathophysiology of diabetic macular edema [8]. They used in silico models to simulate the pathophysiological processes of diabetic macular edema, comprising angiogenesis, inflammation, oxidative stress, and blood–retinal-barrier alteration, and the likely mode of action of intravitreal aflibercept injection. Thus, this study not only helps us understand the disease mechanism but also the mechanism of therapeutics. By providing insights into how aflibercept interacts with various molecular targets, their study offers a pathway for optimizing drug efficacy and minimizing side effects [8].

Today, advanced bioinformatics and ML are redefining the landscape of drug discovery and therapeutic development. They offer the chance to simulate complex biological systems, identify promising compounds, and predict the effects and interactions of drugs. This might significantly reduce the time and cost of drug development before clinical trials. Moreover, by providing a simulated view of disease mechanisms and drug action, artificial-intelligence-derived ML models can further support precision medicine tailoring treatments to patients’ unique molecular profiles.

## 4. Advancements in Disease Risk Prediction and Early Detection

Early detection and risk prediction are central to preventing new disease incidence in healthy individuals as well as the progression of chronic diseases. In this Special Issue, referring to human health and disease, several studies highlight how ML is improving our ability to stratify risk and predict disease onset. Using a range of clinical, molecular, and omics datasets, ML algorithms can identify individuals at high risk before symptoms manifest, paving the way for preventative interventions that can improve patient outcomes and reduce healthcare costs.

As mentioned above, Zhao et al. have examined the relationship between obesity and AD, leveraging ML to explore the genetic polymorphisms—such as those in the APOE gene—that may influence disease susceptibility in the context of metabolic health [7]. This study enhances our understanding of AD and offers potential pathways for early intervention. Moreover, the study conducted by our group identifies key metabolic markers, such as bile acids and ceramides [4]. It demonstrates that these parameters are more important risk predictors, whereas anthropometric features such as age, sex, waist circumference, and body mass index have significantly lower contributions according to a predictive ML model. Thus, the incorporation of these new parameters into risk prediction allows a deeper understanding of metabolic dysregulation in diabetes. It further provides a potential foundation for early preventive strategies and illustrates how ML can reveal complex, disease-associated metabolic patterns whose analysis can aid in preventive care [4]. Similarly, improving diagnostic prediction and identifying biomarkers enabling improved diagnosis and early detection are also addressed in the studies by Luo et al., [1] Akshay et al. [3], and Zhang et al. [2]. In oncology, predictive modeling is similarly advancing early detection efforts. The study by Gil-Rojas et al. further highlights the role of adequate screening. In their study involving patients with hepatocellular carcinoma, they utilized ML to analyze predictors of mortality. They found that the currently widely used alpha-fetoprotein isoform lacks utility as a prognostic factor for mortality. Other variables, in particular the etiology of hepatocellular carcinoma, are more useful and provide more information [9]. Another interesting article in the field of oncology was provided by Behara et al. They have proposed a tool for timely skin cancer identification that will assist oncologists in early diagnosis and treatment, visually explaining the decisions made via ML [10]. 

Such efforts underscore the potential of ML to enhance diagnostics and early detection for many diseases, ultimately leading to more-tailored treatment plans and better patient outcomes.

## 5. Explainability and Transparency in Machine Learning Models

As ML models become more sophisticated, the importance of model interpretability grows, particularly in the healthcare field, where clinicians and patients need to understand the basis of diagnostic and therapeutic recommendations. The complexity of “black-box” ML models, such as deep neural networks, often limits their applicability in clinical settings, where interpretability is essential for trust and informed decision-making. Addressing this challenge, Behara et al., as mentioned above, developed an explainable deep learning model for skin cancer classification [10]. By focusing on transparency in model design, they created an algorithm that not only performs well but also provides interpretable insights into its predictions. It is designed to visualize the decisions made by a neural network model, enhancing both explainability and transparency. This approach bridges the gap between high model accuracy and clinical usability, making it more feasible for deployment in medical workflows. 

The importance of transparency is also evident in the work of Bertin et al., who compared the capacities of several ML tools to evaluate microscope images [11]. In view of known inconsistencies between different laboratories and individual human experts, they aimed to standardize the processing of immunofluorescence images by means of ML and an autonomous analysis. The overall merit of their work lies in their attempt to identify general guidelines, limitations, and possible strategies for future procedures, potentially resulting in greater reliability in diagnosis and medical decision-making and, ultimately, the establishment of better “gold standards”. This has the potential to push medical practice to a new level.

Explainable ML models not only facilitate integration into clinical practice but also improve regulatory approval and patient trust. This Special Issue highlights that while ML models’ complexity may increase with accuracy, maintaining a degree of transparency is paramount, especially when patient health is at stake.

## 6. Challenges and Future Directions

While the achievements highlighted in this Special Issue underscore the promise of ML in healthcare, several technical and practical challenges must be addressed to enable clinical integration. Data quality and consistency across sources are essential for building reliable models, and rigorous validation on diverse patient populations is crucial for ensuring model robustness and applicability. The complexity of many advanced ML models, particularly “black-box” algorithms like deep neural networks, poses another challenge, as interpretability is key for clinical decision-making. Developing models that are transparent, interpretable, and tailored to healthcare contexts will enhance their usability and trustworthiness in clinical settings.

Future research should focus on creating ML models that are not only highly accurate but also easy to interpret and clinically actionable. Interdisciplinary collaboration among data scientists, biologists, and healthcare providers will be essential for aligning model design with real-world needs and clinical standards. As ML applications in healthcare continue to evolve, addressing these practical and technical challenges will be essential to fully realize the potential of ML-driven precision medicine.

## 7. Conclusions

In conclusion, this Special Issue, “Machine Learning and Bioinformatics in Human Health and Disease”, presents a compelling overview of how ML and bioinformatics are driving innovations in precision medicine. The research featured here highlights advancements in multi-omics integration, predictive modeling, and therapeutic strategies, representing significant progress toward more personalized and effective healthcare. However, translating these breakthroughs into routine clinical practice will require constant efforts to ensure model transparency, data quality, and clinical usability. This collection of studies lays a strong foundation for a future where computational methods, enhanced by interdisciplinary collaboration, can improve health outcomes and transform personalized care.
